# A remarkable new cave scorpion of the family Pseudochactidae Gromov (Chelicerata, Scorpiones) from Vietnam

**DOI:** 10.3897/zookeys.71.786

**Published:** 2010-12-14

**Authors:** Wilson R. Lourenço, Dinh-Sac Pham

**Affiliations:** 1 Muséum national d’Histoire naturelle, Département de Systématique et Evolution, Section Arthropodes (Arachnologie), CP 053, 57 rue Cuvier, 75005 Paris, France; 1 Institute of Ecology and Biological Resources (IEBR), Vietnam Academy of Science and Technology (VAST), 18 Hoang Quoc Viet, Cau Giay, Hanoi, Vietnam

**Keywords:** Scorpion, Vietnam, Phong Nha - Ke Bang National Park, karst cave system, new genus and species, troglobitic element

## Abstract

A new genus and species of scorpion belonging to the family Pseudochactidaeare described based on four specimens collected in the Tien Son cave at the Phong Nha - Ke Bang National Park, Quang Binh Province, Vietnam. The new species represents a true troglobitic element, the first one known for the family Pseudochactidae. This represents the third known record of a pseudochactid, and the first from Vietnam.

## Introduction

One of the most remarkable scorpions described during the last 30 years is Pseudochactas ovchinnikovi Gromov, 1998, discovered in an isolated mountainous region of southeastern Uzbekistan and southwestern Tajikistan in Central Asia. Although this scorpion shares some features with buthid and nonbuthid scorpions, it is remarkable because it displays a number of characters unique among recent (extant) scorpions, including a distinct trichobothrial pattern. This led Gromov (1998) to create a new monotypic family, the Pseudochactidae Gromov, 1998.

Subsequently, authors have not reached a consensus regarding the phylogenetic position of this enigmatic scorpion. Based on its peculiar trichobothrial pattern, Fet (2000) suggested a relationship to the most plesiomorphic Buthidae C. L. Koch, 1837 or to Chaerilidae Pocock, 1893. Lourenço (2000) placed Pseudochactas in a new superfamily, Chaeriloidea Pocock, 1893, implying that he considered it to be the sister group of Chaerilus. Although there is widespread agreement that Pseudochactas is basal within recent scorpions, its precise phylogenetic position remains a matter of debate (Fet et al. 2004). In an exhaustive study of Pseudochactas ovchinnikovi, Prendini et al. (2006) concluded that the most plausible position for this ‘living fossil’ would be as the sister-group of Buthidae.

Shortly after these publications, a second genus and species belonging to the family Pseudochactidae, Troglokhammouanus steineri Lourenço, 2007, was described from karst caves in Laos (Lourenço 2007a). This new element of the Pseudochactidae reopened the question about the origins and affinities of this family and led to new biogeographical interpretations (Lourenço 2007a). The precise morphology of this new pseudochactid scorpion was also complemented by SEM studies and a further comparison with elements of the family Chaerilidae (Lourenço 2007b).

Since the description of Troglokhammouanus steineri (Lourenço 2007a, b), no new insights have been published on this subject. While prospecting scorpions in a karst cave system in Vietnam, the second author was able to collect several specimens of a new pseudochactid scorpion. These are described here as a new genus and species. In this note we do not propose new phylogenetic or biogeographical considerations, since these have already been largely discussed by Lourenço (2007a). It is important, however, to notice that the new Vietnamese pseudochactid comes from caves belonging to the same karst system as those in which Troglokhammouanus steineri was found in Laos. This could suggest that this region of Southeast Asia may represent a refuge or an endemic centre for elements of this family. Finally, as suggested by Prendini et al. (2006), the discovery of Pseudochactas ovchinnikovi could represent the most remarkable scorpion discovery during the last (20^th^) century. In this same vein, the discoveries of two new genera and species of pseudochactids in Laos and Vietnam are far from negligible.

## Orogeny and geodynamics of South East Asia

The Southeast Asia or Indochina tectonic plate forms the core of the geological structure of southeastern Asia. This plate comprises the countries of Vietnam, Laos, Cambodia and western Thailand, but according to Metcalfe (2002), also the southeastern portion of the Malayan Peninsula, a fragment of Sumatra, and westernmost portion of Borneo.

The Southeast Asia plate originated during the Proterozoic. It became detached during Palaeozoic and drifted northward. The carbonate platforms were developed during the Devonian-Late Palaeozoic. The Palaeozoic history of detachment and collision is quite speculative. The equivalent of Caledonian orogeny, followed by the formation of the Palaeotethys Ocean is quite possible. Climate records indicate major differences between Sibumasu, Indochina and South China during the Late Palaeozoic. During the Triassic, as a result of the Indosinian orogeny and closure of the Palaeotethys Ocean, the Southeast Asian plate joined the Asian continent (Metcalfe 2002; Senghor and Hsü 1985; Golonka et al. 2006).

**Figure 1.**
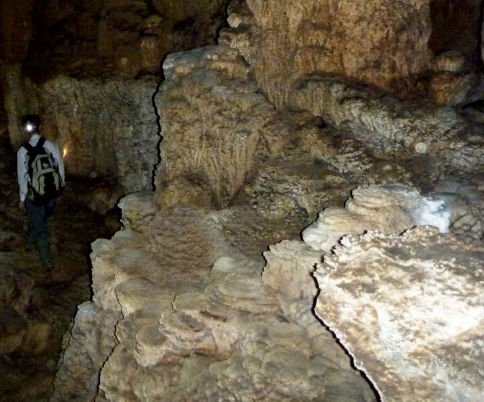
Tien Son Cave, internal view, showing second author searching for scorpions.

## Geology and ecology of the region

In Central Vietnam, the dominant geological feature is the Truong Son Range. This string of mountains and plateaus, also known as the Annamite Mountain Range, is roughly 1200 km long and 50–75 km wide, intersected by passes and lowlands. Most of its hills lie between elevations of 500–2000 m, and for much of its distance they run parallel to the central coastline, straddling the border with Laos. Central Vietnam’s Truong Son Range is a transitional region between the subtropical communities of the North and the tropical ones of the South, and it harbours many endemic species (Groves and Schaller 2000; Herrmann et al. 2002). The Truong Son Range can be divided into three regions: (i) the Northern Truong Son, with much of its region being composed of ancient marine basins, that have been uplifted and now are heavily eroded and form the characteristic sharp karst ridges and peaks with extensive systems of caves, tunnels, and underground rivers and streams; (ii) the Central Truong Son, dominated by the Kon Tum Massif: an enormous, largely granitic formation, which is among the oldest exposed rocks in Southeast Asia; and (iii) the Southern Truong Son, including Vietnam’s remaining uplands with Dac Lac, Da Lat and Di Linh Plateaus, a series of eroded granite and basalt plateaus dotted with isolated peaks. In the Northern Truong Son, Phong Nha - Ke Bang is a region located within the most extensive tracts of limestone karst habitat in Asia. This unique karst system (290–255 My) was likely uplifted in the early Triassic, differs substantially in terms of both geology and habitat from adjacent regions (Sterling et al. 2006; Ziegler 2008; Ziegler and Vu 2009).

**Figure 2.**
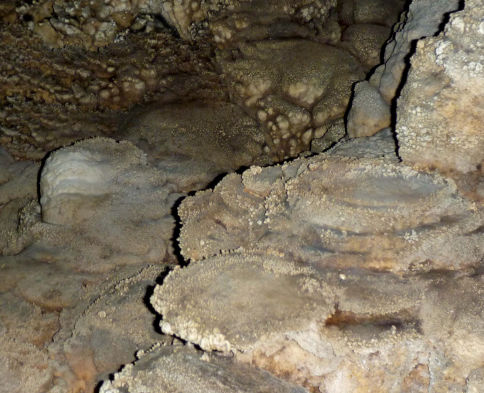
Site in the cave where scorpions were found.

The Phong Nha-Ke Bang karst is the oldest major karst area in Asia. It has been subject to massive tectonic changes and comprises a series of rock types that are interbedded in complex ways. Probably as many as seven different major levels of karst development have occurred as a result of tectonic uplift and changing sea levels, thus the karst landscape of PNKB is extremely complex with high geodiversity and many geomorphic features of considerable significance. There is also strong evidence that sulphuric dissolution and hydrothermal action have played an important role in shaping the general landscape and the caves, though this has not yet been properly assessed.

Modern Phong Nha-Ke Bang is a result of five stages in the Earth’s crustal development and movement: Late Ordovician - Early Silurian Stage (about 450 My), Middle-late Devonian Stage (about 340 My), Carboniferous-Permian (about 300 My), Mesozoic Orogenic stage, and Cenozoic stage (Ziegler and Vu 2009; UNEP-WCMC 2006).

## Location, ecology and climate of the national park and caves

Phong Nha - Ke Bang (Vietnamese: *Vườn quốc gia Phong Nha-Kẻ Bàng*) is now a national park and UNESCO World Heritage Site in the Bố Trạch and Minh Hóa Districts of central Quang Binh Province, in north-central Vietnam, about 500 km south of Hanoi. The park borders the Hin Namno Nature Reserve in the province of Khammouan, Laos (Mouret 2001) in the west, 42 km east of the South China Sea. Phong Nha-Ke Bang National Park is situated in a limestone zone of 2000 km^2^ in Vietnamese territory and borders another limestone zone of 2000 km^2^ of Hin Namno in Laotian territory. The core zone of this national park covers 857.54 km^2^ and a buffer zone covers 1954 km^2^. The park was created to protect one of the world’s two largest karst regions, with 300 caves, and also protects the ecosystem of limestone forest of the Annamite Range region along the north-central coast of Vietnam.

Phong Nha-Ke Bang area is noted for its cave systems with a total length of about 126 km; only 20 caves have been surveyed by Vietnamese and British scientists; 17 of these are located in the Phong Nha area and three in the Ke Bang area. Before discovery of Son Doong Cave, Phong Nha held several world cave records, as it has the longest underground river, as well as the largest caverns and passageways. The park derived its name from Phong Nha cave, the most beautiful of all.

Like northern Central Vietnamin general and Quang Binh Province in particular, the climate in this national park is tropical, hot, and humid. The annual mean temperature ranges from 23 to 25 °C, with extremes of 41°C in the summer and a 6°C in the winter. The hottest months in this region are from June to August, with an average temperature of 28°C, and the coldest months from December to February with an average temperature of 18°C. Annual rainfall is 2000–2500 mm, and 88% of the rainfall occurs from July to December. With more than 160 rainy days per year, no month is without rain. Mean annual relative humidity is 84% in forests.

Tien Son Cave, where the new scorpion was found is located in Son Trach Commune, Bố Trạch District. The entrance is located 1 km from Phong Nha Cave, at an altitude of 200 m. Tien Son Cave is 980 m in length. A 10 m deep hole is situated 400 m from the entrance, after which a 500 m long underground cave is open exclusively to professional scientists. Like Phong Nha Cave, this cave features spectacular stalactites and stalagmites. According to British speleologists, Tien Son Cave was created tens of millions years ago, when a water current holed this limestone mountain in Ke Bang. Following a series of movements of rocks, this mass was levered or lowered, blocking the current and creating what is now Tien Son Cave, while the flow of the underground river was redirected to Phong Nha Cave. Although Phong Nha and Tien Son Caves are located next to each other, there are no passages linking them (UNEP-WCMC 2006).

## Methods

Scorpions were collected by the second author, while exploring the caves with the help of standard electric torches. Scorpions were found under some heavy flat rocks, about 200 m from the main cave entrance. Measurements and illustrations were made using a Wild M5 stereo-microscope with a drawing tube and an ocular micrometer. Measurements follow those of Stahnke (1970) and are given in mm. Trichobothrial notations are those developed by Soleglad and Fet (2001) and the morphological terminology mostly follows that of Hjelle (1990), Prendini et al (2006) and Lourenço (2007a, b).

## Taxonomic treatment

Family Pseudochactidae Gromov, 1998

### Genus Vietbocap gen. n.

urn:lsid:zoobank.org:act:99306155-EE7F-4197-80E4-8DA73E20FAED

#### Diagnosis.

Cheliceral movable finger with three denticles (medial, subdistal, external distal) on dorsal edge; external distal denticle smaller than internal distal denticle. Anterior margin of carapace depressed with a moderate concavity, posterior margin shallowly recurved. Lateral ocelli absent. Pair ofcircumocular sutures with a broad U-shaped configuration (diagnostic for family), only vestigial and incomplete in the posterior region to median ocular tubercle. Median ocelli absent; median tubercle represented by a smooth depressed zone. Anterosubmedial carinae absent from zone limited by circumocular sutures. Type D trichobothrial pattern (Soleglad and Fet 2001, 2003a) with 35 trichobothria per pedipalp: 12 on femur, of which five dorsal, four internal and three external (*d*_1_, *d*_4_, *d*_5_ and *i*_4_ extremely reduced; *i*_4_ absent, in one specimen); 10 on the patella, of which three dorsal, one internal, six external (*est* extremely reduced; absent in one specimen); ventral surface without trichobothria; 13 on the chela, of which five on manus, eight on fixed finger (*est* displaced to cutting edge of fixed finger); pedipalp femur dorsal trichobothria with ‘beta-like’configuration. Sternum pentagonal, type 1 (Soleglad and Fet 2003b), moderately compressed horizontally, markedly longer than wide, external aspect not flat, with a concave region, posteromedian depression round. Telotarsi each with several spinular setae not clearly arranged in rows. Metasomal segment V with a weakly marked pair of ventrosubmedian carinae; no ventromedian carina between ventrosubmedian carinae. Fixed and movable fingers strongly curved; dentate margins each with median denticle row comprising eight oblique granular subrows; internal and external accessory granules at base of each subrow. Respiratory spiracles small, semi-oval. Pro-and retrolateral pedal spurs present on legs I-IV. Tibial spurs absent from all legs.

#### Derivatio nominis:

The generic name is a combination of *Viet* (for Vietnamese) and *bocap* (scorpion in Vietnamese language).

#### Type species:

Vietbocap canhi sp. n.

### Vietbocap canhi sp. n.

urn:lsid:zoobank.org:act:DBD6B87B-36F9-4F09-8584-B31ACB125634

Figs 3 [Fig F4] [Fig F5] [Fig F6] 24 

#### Diagnosis:

as for the genus.

#### Type material:

male holotype; female and two male paratypes. Vietnam, Quang Binh Province, north-central Vietnam, Bố Trạch - Minh Hóa District, Phong Nha - Ke Bang National Park, Tien Son Cave (17°32'N; 106°16'E), mid section of cave (200 m from cave entrance), 16/V/2010 (D.-S. Pham). Holotype and female paratype are deposited in the collection of the Muséum national d’Histoire naturelle, Paris. The other paratypes are deposited in the collections of the Institute of Ecology and Biological Resources, Vietnam Academy of Science and Technology, Hanoi.

**Figsure 3–6.**
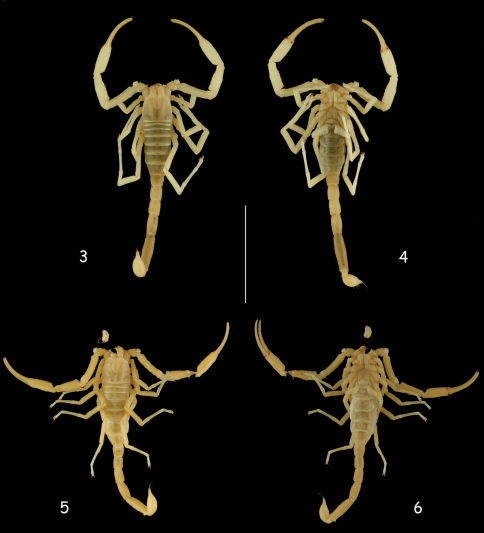
Vietbocap canhi sp. n., male holotype and female paratype, dorsal and ventral aspects. Scale bar = 10 mm.

#### Patronym.

In honour of Dr. Le Xuan Canh, Director of the Institute of Ecology and Biological Resources, Vietnam Academy of Science and Technology, Hanoi, for his support of scorpion research in Vietnam.

#### Description:

based on the male holotype and paratypes (measurements given in mm after the description).

Colour. General coloration yellowish to pale yellow; cheliceral teeth, telson tip and rows of granules on pedipalp fingers reddish-yellow to dark reddish.

Morphology. Chelicerae: dorsal edge of fixed finger, with four denticles (basal, medial, subdistal, distal); ventral edge with 4–5 very reduced denticles; movable finger with three denticles (medial, subdistal, external distal) on dorsal edge, without basal denticles; ventral edge with 4–5 reduced denticles; external distal denticle smaller than internal distal denticle; ventral aspect offingers and manus with numerous macrosetae. Carapace. Anterior margin depressed with a moderately marked concavity. Lateral ocelli absent. Median ocular tubercle represented by a smooth depressed zone; median ocelli absent; interocular furrow obsolete. One pair of vestigial circumocular sutures with a broad U-shaped configuration, incomplete behind median ocular tubercle. Anteromedian and posteromedian furrows shallow; posterolateral furrow shallow, weakly curved; posteromarginal furrow narrow, very shallow. Carapace almost totally smooth, except for some isolated granules anteriorly; acarinate; anterosubmedial carinae absent from the zone internal to circumocular sutures. Pedipalp segments apilose. Femur with five discernible carinae, all weak to vestigial; intercarinal surfaces smooth. Patella with 5–6 discernible carinae; ventrointernal carinae with some spinoid granules; intercarinal surfaces smooth. Chela with only vestigial carinae, rounded and smooth. Fixed and movable fingers strongly curved; dentate margins each with median denticle row comprising eight oblique granular sub-rows; each sub-row comprising several small granules and internal and external accessory granules. Trichobothria:Orthobothriotaxic, Type D (Soleglad and Fet 2001, 2003a), ‘beta-like’configuration, *d*_2_ situated on dorsal surface, *d*_3_ and *d*_4_in same axis of the femur, parallel and closer to dorsoexternal carina than is *d*_1_,angle formed by *d*_1_, *d*_3_and *d*_4_ opening toward internal surface; totals: femur, 12 (five dorsal, four internal, three external); patella, 10 (three dorsal, one internal, six external); chela, 13 (five manus, eight fixed finger). Legs I to IV: tibiae, without spurs; basitarsi each with a pair of pro- and retrolateral spurs; telotarsi each with several spinular setae, not well arranged in rows. Sternum pentagonal, type 1 (Soleglad and Fet 2003b), moderately compressed horizontally, markedly longer than wide, external aspect not flat, with a concave region, posteromedian depression round. Pectines each with 3–4 distinct marginal lamellae in male and female, 8–9 well-delineated median lamellae present in male (7 in female). Fulcra absent or vestigial. Pectinal tooth count: 9–9 in males and 7–7 in female. Genital operculum completely divided longitudinally; genital plugs observed in male. Mesosoma:pre-tergites smooth and shiny; post-tergites II-VI smooth, apart from some minute granules; VII with a few granules and a pair of dorsosubmedian and dorsolateral carinae, reaching posterior edge of segment. Sternites almost entirely smooth, acarinate; surfaces with scattered macrosetae; distal margins with sparse row of macrosetae; respiratory spiracles small, semi-oval in shape. Metasoma covered in short macrosetae. Ten carinae on segments I to III; eight carinae on segment IV; four on segment V. Dorsosubmedian carinae moderately developed on segments I-IV, absent on segment V; spinoid granules absent. Other carinae moderately to weakly developed on segments I-V. Telson long and slender; vesicle smooth on all faces; aculeus shorter than vesicle and weakly curved, without a subaculear tubercle ventrally. Form of venom glands unknown.

**Figsure 7–11. F4:**
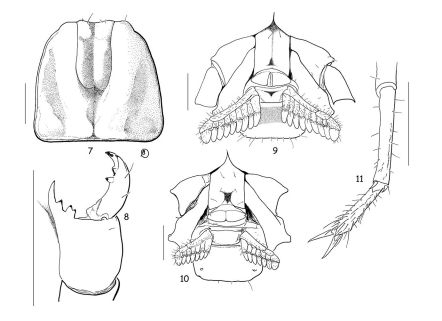
Vietbocap canhi sp. n. (M=male, F=female). **7** Carapace, dorsal aspect (M) **8** Chelicera, dorsal aspect (F) **9–10** Ventral aspect, showing sternum, genital operculum, pectines and sternite III (M & F) **11** Leg IV, showing absence of tibial spur and telotarsi with spinular setae (F). Scale bars = 1 mm.

**Figsure 12–15. F5:**
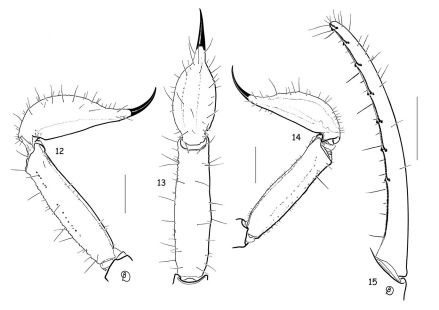
Vietbocap canhi sp. n. (M=male, F=female). **12–13** Metasomal segment V and telson, lateral and ventral aspects (M) **14** Idem female **15** Movable finger of pedipalp chela with subrows of granules (M). Scale bars = 1 mm.

**Figures 16–18. F6:**
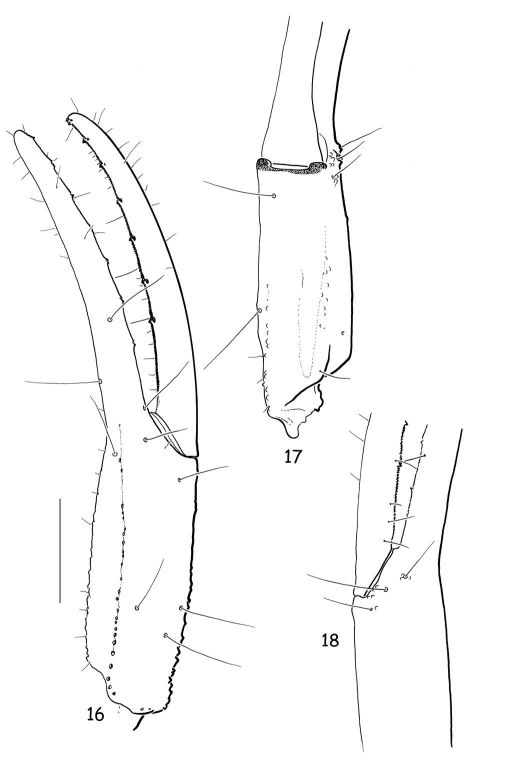
Vietbocap canhi sp. n., female paratype. Trichobothrial pattern. Chela, dorso-external, ventral and internal aspects. Scale bar = 1 mm.

**Figures 19–24.**
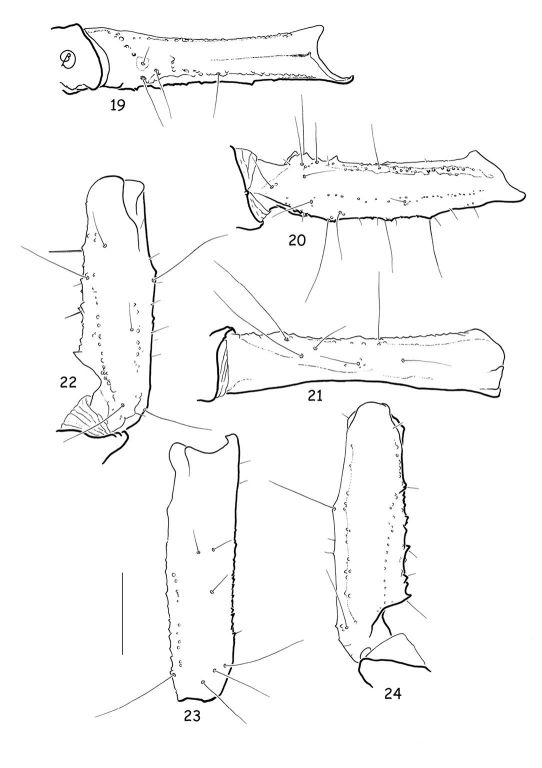
Vietbocap canhi sp. n., female paratype. Trichobothrial pattern. **19–21** Femur, internal, dorsal and external aspects **22–24** Patella, dorsal, external and ventral aspects. Scale bar = 1 mm.

#### Geographic distribution.

Only known from the type locality.

#### Measurements (in mm) of male holotype/female paratype.

Total length 22.4/21.3. Carapace: length 2.9/2.8; anterior width 2.0/1.8; posterior width 3.2/2.9. Mesosoma length 5.5/6.4. Metasomal segments: I, length 1.2/1.0, width 1.4/1.2; II, length 1.4/1.2, width 1.3/1.0; III, length 1.5/1.4, width 1.2/0.9; IV, length 2.1/1.7, width 1.1/0.8; V, length 3.9/3.2, width 1.1/0.8, depth 0.9/0.8. Telson length 3.9/3.6; vesicle length 2.4/2.2, width 1.3/1.0, depth 1.2/0.9. Pedipalp: femur length 3.8/3.1, width 0.9/0.7; patella length 3.6/3.2, width 1.1/0.9; chela length 7.1/5.8, width 1.2/1.0, depth 1.0/0.9; movable finger length 4.2/3.9.

#### Key to the known genera and species of Pseudochactidae.

**Table:** 

1	Median and lateral ocelli present; leg tibial spurs present	**3**
2	Median and lateral ocelli absent; leg tibial spurs absent	Vietbocap canhi**sp. n.**
3	Circumocular sutures incomplete; peg sensillae of pectines rounded	Troglokhammouanus steineri Lourenço, 2007
–	Circumocular sutures complete; peg sensillae of pectines spatular	Pseudochactas ovchinnikovi Gromov, 1998

## Supplementary Material

XML Treatment for Vietbocap

XML Treatment for Vietbocap canhi
